# Immunological response enhancement in cows with subclinical mastitis fed diet supplemented with *Macleaya cordata*

**DOI:** 10.3389/fvets.2025.1503487

**Published:** 2025-04-15

**Authors:** Mostafa S. A. Khattab, Li Haiqiang, Tang Shaoxun, Yan Qiongxian, Liu Yong, Tan Zhiliang, Lu Qi

**Affiliations:** ^1^State Key Laboratory of Forage Breeding-by-Design and Utilization, National Engineering Laboratory for Pollution Control and Waste Utilization in Livestock and Poultry Production, and Hunan Provincial Key Laboratory of Animal Nutritional Physiology and Metabolic Process, Institute of Subtropical Agriculture, Chinese Academy of Sciences, Changsha, China; ^2^Dairy Department, National Research Centre, Giza, Egypt; ^3^College of Animal Science, Guizhou University, Guiyang, China; ^4^College of Advanced Agricultural Sciences, University of Chinese Academy of Sciences, Beijing, China

**Keywords:** antioxidant capacity, immune indices, *Macleaya cordata*, mastitis, milk yield

## Abstract

**Introduction:**

The present study explored the immune response, milk production and health status of mastitis-infected lactating cows fed diets supplemented with Macleaya cordata extract.

**Methods:**

Twenty-four Holstein and Jersey cows were equally assigned to two experimental groups: the first group was fed a control diet (control), and the second experimental group was fed a control diet plus Macleaya extract at 8 g/head/d (Macleaya). The experiment was conducted for 60 days. The daily milk yield was recorded, and the milk samples were analyzed for total solids, fat, protein, and lactose contents.

**Results:**

Blood samples were analyzed for different blood constituents, biochemical parameters, antioxidant capacity and immune indices. Compared with the control, the addition of Macleaya improved immune indices (*p* < 0.05). No significant differences (*p* > 0.05) were recorded between the two groups for different rumen liquor parameters, antioxidant capacities, milk yields or compositions. However, supplementing the diet with Macleaya significantly decreased SCC, SAA, and endotoxin.

**Conclusion:**

This study suggested that supplementing the diets of lactating cows with Macleaya extract potentially improved their immune competence without adversely impacting their productive performance.

## Introduction

Mastitis in cows is an inflammatory reaction of the mammary gland udder tissue caused by either physical injury or microbial infection. It is regarded as the most prevalent disease that causes financial losses for the dairy industry because of lower milk quality and output (1, 2). On average, the overall failure cost caused by bovine mastitis is projected to be $147 per cow per year, with milk production losses and culling accounting for 11% to 18% of the gross margin per cow per year (3). Seventy percent of the overall losses are due to mammary tissue injury, which causes reduced milk output (4). Clinical, subclinical, and chronic mastitis are the three categories into which bovine mastitis can be divided according to the severity of inflammation. A fever and a red, swollen udder are two obvious signs of a clinical case of bovine mastitis that are simple to identify. The cow milk seems watery, with flakes and clots (5).

In dairy cows, mastitis is caused by bacteria that enter the teat canal and mammary glands and cause inflammation of the udder. These bacteria grow and create toxins that injure milk-secreting tissue, in addition to causing physical damage and chemical irritation. These factors lead to an increase in leukocytes, or somatic cells, in milk, which decreases their amount and degrades the quality of both the milk and its byproducts. Seventy percent of the losses are attributable to injury to the mammary tissue, which results in reduced milk production (4). The incidence of mastitis in a herd can be reduced, but mastitis cannot be completely eradicated. Good husbandry and sanitation techniques, teat dipping after milking, treating mastitis during the nonlactating phase, and culling chronically sick animals are the main components in the management of mastitis (5).

The two main strategies for controlling mastitis are to either reduce the teat’s exposure to possible pathogens or increase the ability of dairy animals to fight infection. Widely prevalent in southern China, *Macleaya cordata* (MC) is a perennial herb belonging to the group *Papaveraceae* that is used in traditional Chinese medicine (6). The primary bioactive components of MC extract (MCE) are alkaloids, primarily sanguinarine and chelidonine, which have been shown to have antibacterial (7), anti-inflammatory (8), antitumor (9), and antioxidant properties (10).

In the European Union (EU) and China, sanguinarine derived from MC has been authorized for use as a feed supplement. Research has shown that this substance enhances the immune system and growth performance of livestock (11, 12). The majority of MCE research to date has been conducted on monogastric animals. For example, weaning piglets fed with 50 mg/kg MCE improved growth performance and resulted in a lower rate of diarrhea because the treatment increased the serum IgG concentration, antioxidant activity, and profile of beneficial bacteria in the intestine (13). Yang et al. (14) reported that adding 0.3 g/d MCE to the meals of early-weaned goats reduced the inflammatory response in their lower guts. Adding MCE to the sheep diet enhances their immune system, antioxidant levels, and growth performance (15). To the best of our knowledge, little research has been conducted on the consequences of feeding lactating cows MCE. Consequently, the aim of the current study was to examine the effects of supplementing the diets of lactating cows with dietary *Macleaya cordata* on the immune response, rumen fermentation, and milk yield and composition.

## Materials and methods

All the experimental protocols for the use of animals and the experimental procedures in this study were approved by the Laboratory Animal Welfare and Animal Experimental Ethical Inspection Committee, Institute of Subtropical Agriculture, the Chinese Academy of Sciences, Changsha, China.

Twenty-four Holstein and Jersey cows with similar parities (2 parities), calving dates, daily milk yields, mid-lactation rates and recessive mastitis (somatic cell count (SCC) greater than 200 × 10^3^/mL) were selected for the current experiment. Using a completely randomized block design, the experimental animals were divided into two groups with 12 animals (three Holstein and nine Jersey cows) in each group, and the milk SCCs in the control and experimental groups were 480 × 10^3^/mL and 492 × 10^3^/mL, respectively. The experimental diet was offered to meet their nutrient requirements according to NRC (16) recommendations. The cows in the control group were fed a basic diet, as shown in Table 1 (control), and the cows in the experimental group (Macleaya) were fed a basic diet supplemented with Macleaya extract at 8 g/head/d. The active ingredient of macleaya is benzenoid alkaloids (sanguinarine content of 1.5% and chelidonine content ≥ 0.75%).

**Table 1:** Ingredients and chemical composition of the control diet.

Item	Basic diet
Ingredients, g/kg of DM	
Silage corn	487.80
Spanish alfalfa	48.70
Domestic oat grass	91.50
Timothy	24.40
Beet pulp	24.40
Brewer’s grains	146.30
Molasses	24.40
Corn	79.70
Soybean meal	27.40
Extruded soybean	1.10
Cottonseed meal	15.20
DDGS	15.20
Sodium bicarbonate	1.50
Premix	17.50
Chemical composition, g/kg of DM	
Dry matter (DM)	568.70
Crude protein (CP)	135.20
Ether extract (EE)	55.90
Neutral detergent fiber (NDF)	308.10
Acid detergent fiber (ADF)	176.20
NE_L_ (MJ/kg)	6.70

Each kg of premix contained 9,000 mg CuSO4, 300 mg FeSO4·H2O, 300 mg ZnSO_4_·H_2_O, 31,000 mg CoCl_2,_ 90 mg Na_2_SeO_3,_ 130 mg VA 900,000 IU, 180,000 IU VE 25,000 mg, and 22,000 mg niacin. The nutrient contents were measured, except for the NE_L_.

The experiment lasted for 60 days, including 10 days for adaptation and 50 days as the experimental phase. The animals were raised in individual pens, fed twice daily (06:00 and 13:30), and had free access to clean drinking water.

### Sample collection

#### Feed sample collection

Approximately 500 g of TMR sample was collected and dried in an oven at 65°C for 48 h. Feed samples were stored at −20°C until further analysis.

#### Rumen liquor sample collection

Representative samples of rumen liquor were collected from the animals at 60 days of the experiment. The samples were collected from the central rumen at 4 h after the morning feeding through the oral cavity via an oral stomach tube as reported by Wang et al. (17). In summary, the first 150 mL of collected rumen mixture was discarded, and then the remaining 150 mL was collected and filtered through four gauze layers while being continuously exposed to carbon dioxide.

#### Blood sample collection

On the last day of the experiment, blood samples were collected from the jugular vein of the cows at 4 h post morning feeding. Tubes containing the anticoagulant heparin sodium and a venous blood collection needle were used to collect blood samples from the cows. The tubes of collected blood were inverted gently many times to ensure that the blood sample and anticoagulant were fully mixed. The blood samples were centrifuged for 15 min at 3000× g to quickly separate the plasma. After that, the plasma was separated into sterile 1.5 mL Eppendorf tubes and stored at −80°C.

#### Milk yield recording and sampling

The daily milk yield of each experimental cow was recorded during the 60-day data collection period. Cows were milked three times daily at 05:30, 13:00, and 19:00 h. Milk samples were collected at 60 days, and morning and afternoon milk samples were stored at 4°C until the evening samples were collected. The samples were subsequently pooled according to morning, afternoon, and evening milk yields.

### Sample analysis

#### Feed sample analysis

The feed samples were analyzed for DM (method 930.15), crude protein (CP; method 954.01) and ether extract (EE; method 920.39) content according to the AOAC official methods (18). Neutral detergent fiber (NDF) content was determined according to the procedure of Van Soest et al. (19) without the use of alpha-amylase but with sodium sulfite. Acid detergent fiber (ADF) was analyzed according to AOAC (18) (method 973.18).

#### Rumen liquor sample analysis

pH and the concentrations of ammonia nitrogen (NH_3_-N) and total volatile fatty acid and their proportion (VFA) in the rumen liquor were measured via methods described by Wang et al. (17).

#### Blood sample analysis

Blood samples were used to analyze biochemical parameters, antioxidant parameters, and immune indices. Blood cells [such as the white blood cell (WBC), neutrophil (Neu), lymphocyte (Lym), monocyte (Mon), eosinophil (Eos), basophilic granulocyte (Bas), red blood cell (RBC), hemoglobin (HGB), hematocrit (HCT), mean corpuscular volume (MCV), mean corpuscular-hemoglobin (MCH), mean corpuscular-hemoglobin concentration (MCHC), red blood cell distribution width variable coefficient (RDW-CV), red blood cell distribution width (RDW-SD), platelet count (PLT), mean platelet volume (MPV), platelet distribution width (PDW), and thrombocytocrit (PCT)], and EDTAK 2 anticoagulant whole blood collected within 2 h were tested via an automatic veterinary blood cell analyzer (BC-5000, Mindray, China), and the specific operation was carried out according to the operation procedure of the automatic veterinary blood cell analyzer.

The plasma total protein (TP), albumin (ALB), alanine transaminase (ALT), aspartate transaminase (AST), alkaline phosphatase (ALP), gamma glutamyl transpeptidase (GGT), cholinesterase (CHE), direct bilirubin (DBIL), total bilirubin (TBIL), triglyceride (TG), total cholesterol (CHOL), low-density lipoprotein cholesterol (LDL-c), high-density lipoprotein cholesterol (HDL-c), glucose (GLU), amylase (AMS), D-lactic acid (LAC), lactate dehydrogenase (LDH), blood urea nitrogen (BUN), creatinine (CRE), and calcium (Ca) and phosphorus (P) concentrations were measured on a Mindray BS-230 automatic chemistry analyzer (Shenzhen, China) (20).

For the immune indices, the plasma concentrations of interleukin 1β (IL-1β), interleukin 2 (IL-2), interleukin 6 (IL-6), interleukin 8 (IL-8), tumor necrosis factor (TNF-*α*), immunoglobulin G (IgG), immunoglobulin A (IgA), and immunoglobulin M (IgM) were determined with a Multimode Microplate Reader (Infinite M200 PRO Multimode, Tecan, Männedorf, Switzerland) and a commercial ELISA kit (CUSABIO, Wuhan, China) according to the manufacturer’s protocols.

The samples were analyzed for different antioxidant parameters via assay kits (ELISAs) according to the manufacturers’ instructions. The total antioxidant capacity (TAC), malondialdehyde (MDA), superoxide dismutase (SOD), and glutathione peroxidase (GPx) contents were determined as described by Khattab et al. (21).

#### Milk sample analysis

Milk samples were analyzed for fat, lactose, protein, nonfat solid (NFS), and total solids (TS) contents via a Foss Milko Scan FT1 Analyzer (Foss Electric A/S, Hillerod, Denmark). The average yield (g/d) of each milk component was calculated for individual animals by multiplying the milk yield by the component concentration (g/kg) of milk. The serum amyloid A (SAA) concentration in milk samples was determined via specific ELISA kits, and the endotoxin concentration was determined with a Multimode Microplate Reader (Infinite M200 PRO Multimode, Tecan, Männedorf, Switzerland) and a commercial ELISA kit (CUSABIO, Wuhan, China) according to the manufacturer’s protocols. Energy-corrected milk (ECM) was calculated according to Sjaunja et al. (22).

### Statistical analysis

All the data were subjected to analysis using ANOVA via the MIXED procedure of SAS (version 9.4; SAS Inc.). The fixed effects of breed, treatment, and the interaction effect between breed and treatment were included in the model. The cows represented a random effect. The results are expressed as the means. A *p*-value < 0.05 was considered to indicate a statistically significant difference.

## Results

### Rumen fermentation profile

The effects of supplementation of lactating cows’ diets with *Macleaya cordata* on rumen fermentation profile are presented in Table 2. All values of the different parameters were within the normal range for each parameter (23). The acetate, A/P ratio, isobutyrate, butyrate, and isovalerate concentrations were not significantly greater (*p* > 0.05) in the Macleaya group than in the control group. Additionally, insignificant (*p* > 0.05) reductions in the NH_3_-N, propionate and TVFA concentrations were detected in the Macleaya group compared with those in the control group.

**Table 2:** Effect of supplementing the diets of lactating cows with *Macleaya cordata on* ruminal fermentation.

Item	Control	Macleaya	±SE	*p*-value
pH	6.50	6.56	0.037	0.322
NH_3_-N (mM/L)	6.17	5.39	0.364	0.276
Acetate %	66.40	66.90	0.22	0.318
Propionate %	19.00	18.30	0.14	0.211
A/P ratio	3.51	3.68	0.040	0.202
Iso-butyrate %	0.98	0.94	0.040	0.615
Butyrate %	11.80	11.90	0.15	0.804
Iso-valerate %	0.98	1.03	0.049	0.243
Valerate %	1.33	1.32	0.022	0.886
TVFA mM/L	102.40	86.20	2.26	0.176

Control, control group fed a basic diet; Macleaya, Macleaya group fed a basic diet supplemented with Macleaya extract at 8 g/head/d; ±SE, standard error.

### Plasma biochemical values

Table 3 shows the values of the plasma biochemical parameters of the experimental groups. All values of the different parameters were within the normal range of each parameter. No significant differences (*p* > 0.05) in different plasma biochemical parameters were noted between the experimental groups. Compared with the control group, the Macleaya group tended to decrease (*p* = 0.09) the concentration of CHE and slightly increased (*p* > 0.05) the globulin, ALT, AST, GGT, LDH, BUN, CRE, CHOL, HDL-c, amylase, LDL-c and phosphorus concentrations.

**Table 3:** Effects of supplementing the diets of lactating cows with *Macleaya cordata on* plasma biochemical parameters.

Item	Control	Macleaya	±SE	*p*-value
TP (g/L)	79.30	79.10	0.95	0.343
ALB (g/L)	45.40	44.00	0.57	0.791
Globulin (g/L)	33.80	35.10	0.57	0.190
A/G ratio	1.36	1.27	0.024	0.357
ALT (IU/L)	24.50	32.00	0.63	0.209
AST (IU/L)	80.90	89.10	2.46	0.742
ALP (IU/L)	51.20	49.80	3.50	0.814
GGT (IU/L)	36.80	36.80	1.12	0.957
LDH (IU/L)	1,186	1,193	19.0	0.467
BUN (mmol/L)	5.23	5.82	0.095	0.570
CRE (μmol/L)	51.90	55.40	1.21	0.641
GLU (g/L)	3.71	3.78	0.059	0.637
TG (mmol/L)	0.29	0.26	0.007	0.316
CHOL (mmol/L)	4.25	5.16	0.122	0.231
HDL-c (mmol/L)	3.51	4.37	0.109	0.146
AMS (U/L)	31.10	31.80	0.74	0.433
LDL-c (mmol/L)	1.30	1.83	0.071	0.584
LACT (mmol/L)	2.24	1.89	0.276	0.301
CHE (g/L)	183	164	4.00	0.099
Ca (mmol/L)	2.48	2.15	0.023	0.390
P (mmol/L)	1.88	2.08	0.028	0.667

Control, control group fed a basic diet; Macleaya, Macleaya group fed a basic diet supplemented with Macleaya extract at 8 g/head/d; ±SE, standard error.

### Blood constituents

Table 4 shows the values of blood constituents for the experimental groups. No values of different blood constituents were outside the normal range. The white blood cell count, neutrophil count, lymphocyte count, monocyte count, basophil count, RBC count, HGB, HCT, MCHC, RDW-CV, PLT, MPV, and PCT did not significantly differ between the Macleaya and control groups (*p* > 0.05). Compared with the control group, the Macleaya group presented a significant reduction in Eosinophil. However, compared with the control diet, supplementation with the Macleaya diet significantly increased the MCV, MCH, RDW-SD, and PDW.

**Table 4:** Effect of supplementing the diets of lactating cows with *Macleaya cordata on* blood constituents.

Item	Control	Macleaya	±SE	*p*-value
White blood Cells (10^9^/L)	7.32	7.31	0.261	0.983
Neu (10^9^/L)	3.13	3.09	0.157	0.905
Lym (10^9^/L)	3.46	3.60	0.131	0.605
Mon (10^9^/L)	0.37	0.38	0.044	0.837
Eos (10^9^/L)	0.34	0.22	0.044	0.009
Bas (10^9^/L)	0.02	0.02	0.006	1.000
Neu (%)	42.00	42.30	1.17	0.903
Lym (%)	48.00	49.50	1.14	0.513
Mon (%)	5.13	5.06	0.237	0.881
Eos (%)	4.54	2.85	0.261	0.001
Bas (%)	0.34	0.29	0.092	0.589
RBC (10^12^/L)	5.81	5.73	0.150	0.776
HGB (g/L)	106	111	2.6	0.355
HCT (%)	28.50	30.20	0.76	0.266
MCV (fL)	49.10	53.00	0.58	0.0003
MCH (pg)	18.20	19.50	0.21	0.002
MCHC (g/L)	371	367	1.6	0.260
RDW-CV (%)	21.90	23.10	0.33	0.065
RDW-SD (fL)	38.20	43.60	0.85	0.001
PLT (10^9^/L)	424	314	29.6	0.063
MPV (fL)	5.84	5.81	0.069	0.822
PDW	15.10	15.40	0.07	0.007
PCT (%)	0.25	0.18	0.038	0.068

Control, control group fed a basic diet; Macleaya, Macleaya group fed a basic diet supplemented with Macleaya extract at 8 g/head/d; ±SE, standard error.

### Plasma antioxidant capacity

The antioxidant capacity data are shown in Table 5. The levels of the antioxidant parameters total antioxidant capacity (TAC), superoxide dismutase (SOD), malondialdehyde (MDA) and glutathione peroxidase (GPx) did not significantly (*p* > 0.05) differ between the Macleaya group and the control group.

**Table 5:** Effects of supplementing the diets of lactating cows with *Macleaya cordata on* plasma antioxidant parameters.

Item	Control	Macleaya	±SE	*p*-value
TAC (U/mL)	0.96	0.96	0.019	0.898
SOD (U/mL)	71.20	73.10	2.04	0.637
MDA (nmol/mL)	4.20	3.67	0.278	0.339
GPx (U/L)	442	461	32.9	0.766

Control, control group fed a basic diet; Macleaya, Macleaya group fed a basic diet supplemented with extract at 8 g/head/d; ±SE, standard error.

### Immune indices

The immune indices of the different experimental animal groups are listed in Table 6. The results revealed significant decreases in the levels of IL-1β (*p* = 0.020), IL-6 (*p* = 0.0004), IL-8 (*p* = 0.008), and TNF-*α* (*p* = 0.007) in the Macleaya group compared with those in the control group. However, ceruloplasmin (*p* = 0.015) was greater in the Macleaya group than in the control group. IL-2, heptoglobin, IgA, IgG, and IgM levels were not significantly different (*p* > 0.05) between the experimental groups.

**Table 6:** Effects of supplementing the diets of lactating cows with *Macleaya cordata on* immune indices.

Item	Control	Macleaya	±SE	*p*-value
IL-1β (pg/mL)	53.40	35.50	7.42	0.020
IL-2 (pg/mL)	257	357	174.4	0.570
IL-6 (pg/mL)	145.40	96.40	12.92	0.0004
IL-8 (pg/mL)	139	104	7.40	0.008
TNF-α (pg/mL)	95.30	58.20	6.91	0.007
Haptoglobin (μg/mL)	691	657	34.71	0.317
Ceruloplasmin (μg/mL)	815	1,087	107	0.015
IgA (μg/mL)	1837	1859	56.9	0.793
IgG (μg/mL)	12,811	12,969	41.1	0.740
IgM (μg/mL)	952	921	146.6	0.540

Control, control group fed a basic diet; Macleaya, Macleaya group fed a basic diet supplemented with Macleaya extract at 8 g/head/d; ±SE, standard error.

### Milk yield and composition

The milk yield did not significantly (*p* > 0.05) differ between the Macleaya and control groups (Table 7). However, the ECM was significantly greater in Macleaya than in the control (*p* = 0.0203). Additionally, no significant differences in pH, milk total solids content, milk fat content, milk protein content, milk lactose content, or milk NFS were recorded between the experimental groups. The same trend was observed for milk total solids yield, milk fat yield, milk protein yield, milk lactose yield, and milk NFS yield.

**Table 7:** Effect of supplementing the diets of lactating cows with *Macleaya cordata on* milk yield and composition.

Item	Control	Macleaya	±SE	*p*-value
Milk Yield (kg)	20.27	20.34	0.29	0.22
ECM (kg)	21.80	22.50	0.29	0.0203
pH	6.59	6.60	0.788	0.797
SCC (10^3^/ml)	446	391	85.2	0.048
Milk composition (%)				
TS	13.90	14.40	0.187	0.246
Fat	4.73	5.37	0.241	0.120
Protein	3.99	4.01	0.172	0.768
Lactose	4.51	4.38	0.083	0.297
NFS	9.31	9.20	0.070	0.617
Urea (mg/dL)	19.40	20.00	0.19	0.520
SAA (μg/ml)	9.70	5.84	0.430	0.001
Endotoxin (EU/ml)	12.40	8.04	0.535	0.001
Milk yields (g/d)				
TS	2,383	2,447	88.3	0.713
Fat	797	880	35.4	0.242
Protein	693	685	27.9	0.879
Lactose	796	786	36.9	0.898
NFS	1,625	1,607	66.2	0.889

Control, control group fed a basic diet; Macleaya, Macleaya group fed a basic diet supplemented with Macleaya extract at 8 g/head/d; ±SE, standard error.

Compared with the control diet, the diets supplemented with Macleaya significantly decreased the SCC (*p* = 0.048), SAA (*p* = 0.001) and endotoxin content (*p* = 0.001).

## Discussion

The most expensive disease that affects dairy cattle is still mastitis. When the udder becomes inflamed and germs infiltrate the teat canal and mammary glands, dairy cows develop mastitis. These bacteria grow and release toxins, in addition to physical stress and chemical irritants, which harm milk-secreting tissue.

These factors lead to an increase in leukocytes, or somatic cells, in milk, which decreases its volume and has a negative effect on the quality of milk and its byproducts (5). Mastitis in dairy cattle can be decreased by monitoring somatic cell levels and quickly diagnosing and treating the condition.

As previously stated, the emergence of antibiotic-resistant pathogen strains has become a significant obstacle in antibiotic treatment, and if the use of antibiotics as the primary treatment strategy is still in place, its efficacy is restricted (24, 25). Moreover, the milk industry is being pressured to use fewer antimicrobial medications due to growing concerns about antibiotic resistance in public health issues. Consequently, it is necessary to look for alternatives to antibiotic therapy, particularly those originating from natural sources such as plants and animals (26–28). Researchers are becoming more interested in the use of plants to treat bovine mastitis since they are rich sources of chemicals for traditional medicine. The benefit of plant-derived chemicals over antibiotics is that they do not cause resistance, even after extended use. The low toxicity of chemicals originating from plants is another benefit (2).

Chinese people have long utilized plants of this genus for their diverse medicinal properties. Traditionally, the entire plant of *Macleaya cordata* has been used as a popular medicine to cure inflamed wounds and stings and to temporarily reduce muscular discomfort. *M. cordata* leaves are often used in traditional Chinese medicine to treat rheumatism, scabies, bruises, ulcers, and other skin conditions (29). Both Macleaya species are used primarily for the treatment of inflammation and certain skin conditions. Currently, Trichomonas vaginalis, rheumatoid arthritis, wounds, and other conditions are treated with *M. cordata* in traditional Chinese medicine (30). The anticancer, antiseptic, anti-inflammatory, and insecticidal properties of several extracts and isolated parts of the Macleaya species have been assessed (31). According to previous studies (32–35), quaternary benzo[c]phenanthridine alkaloids are the main bioactive substances. Numerous pathophysiological mechanisms that respond to tissue injury and the host’s defense against invasive microorganisms depend on inflammation. An increase in cytokines and nitric oxide production is indicative of inflammation. Numerous investigations have indicated that the Macleaya species has an anti-inflammatory effect (36). According to Vrba et al. (37), the anti-inflammatory properties of sanguinarine and chelerythrine may also be linked to their capacity to prevent the production of superoxide radicals by phagocyte NADPH oxidase. However, research on the direct effects of Macleaya extracts on mastitis in dairy cows is limited. These findings suggest that Macleaya has potential effects on oxidative stress control and that it has immune-boosting capabilities, antioxidant properties, antibacterial effects, and anti-inflammatory properties. However, the specific impact on mastitis remains to be fully explored.

The current study showed no noticeable effect of *Macleaya cordata* extract on rumen fermentation profile of cows. These findings might be due to the bioactive alkaloids in *Macleaya cordata*, such as sanguinarine and chelerythrine, which known for their antimicrobial and anti-inflammatory properties (38). While these compounds may affect certain microbial populations, they might not target the specific microbes involved in producing key fermentation metabolites (e.g., VFAs, NH_3_-N) (39). Also, the rumen microbiome is remarkably adaptable and robust.

Leucocyte settling is the mammary gland’s main defensive function during an inflammatory response. These typically have fewer than 200,000 cells per milliliter of milk. The cell count increases when mastitis is present, mostly due to leukocyte infiltration. Inflamed neutrophils and macrophages eliminate cellular aggregates and phagocytize ingested bacteria (40, 41). An aspect of nonspecific resistance, the acute phase response is characterized by a rapid rise in protein synthesis, which is known to be a hallmark of inflammation in animals (42, 43).

The findings of the present study revealed that the addition of macleaya extract to the diet decreased the IL-1β, IL-6 and IL-8 levels. A reduction in the IL-1β, IL-6 and IL-8 levels in cows fed Macleaya extract may indicate the resolution of inflammation or successful treatment for mastitis. Cows with naturally occurring and experimentally induced mastitis, as well as those injected with bacterial LPS, have been shown to have relatively high levels of IL-6 in their blood and milk (44, 45). The fact that IL-6 may stay in the bloodstream longer than other proinflammatory cytokines is a very significant benefit (46).

Both clinical and subclinical diseases may develop from mastitis. According to recent research, the host’s reaction during the early stages of illness, including the impacts of cytokines released, influences the consequences of infection (47–49). Small molecule proteins called cytokines are crucial for intercellular communication. Numerous processes, including cell differentiation, proliferation, remodeling, degeneration, regeneration, and even cell death, are either driven or hindered by cytokines. The SCC and levels of generated cytokines (interleukin (IL)-1, IL-2, IL-4, IL-5, IL-6, IL-8, IL-10, and IL-12) in milk increase in both naturally occurring and experimentally induced mastitis (50). One of the cytokine families that is most commonly represented is the IL-1 family (51). Through a number of signaling pathways, IL-1 family cytokines interact with immune competent tissue cells (52), which mostly carry out inflammatory, autoimmune, fracture-initiating, and mitogenic tasks. Interleukin-1 alpha (IL-1α) locally regulates first-line defense mediators in experimental acute bovine mastitis, including prostaglandin (PG-F2*α*) and leukotriene (LT-B4), indicating its participation in early inflammation (53). T and B lymphocytes, eosinophils, basophils, mast cells, plasma cells, and epithelial cells are the primary producers of IL-4 in the mammary glands of cows (54, 55). These cells also serve as the foundation for type 2 immunity (56). IL-1 has an indirect effect on immunity by interacting with other messenger molecules through a number of signaling pathways (52). Macrophages, lymphocytes, monocytes, endothelial cells, and fibroblasts in the mammary gland generate IL-1. Furthermore, IL-1 promotes the synthesis of TNF-*α*, IL-6, IL-8, and IL-12 in addition to its own (55).

Numerous experimental investigations have demonstrated the importance of inflammatory components in the initial inflammatory response. Upon identifying microbial components, bovine mammary epithelial cells can trigger an innate immune response through the release of proinflammatory cytokines (IL-1β, TNF-*α*, IL-6, and IL-8) and antimicrobial molecules (cathelicidin and defensins) (57, 58). The primary source of the proinflammatory cytokine tumor necrosis factor (TNF)-*α* is produced by macrophages. It may carry out a variety of tasks, including inducing inflammatory responses and encouraging the manufacture of other cytokines, regulating essential cell activities, and preserving tissue homeostasis, depending on where it is released and which receptor it binds to (59–61). The acute-phase cytokine TNF-α is generated in the early phases of infection (62, 63). The TNF-α concentration significantly decreased as the cows recovered (64). Consequently, the significance of TNF-α can extend beyond its proinflammatory function. However, with endotoxin infusion, the TNF-*α* concentration only slightly increased (65). TNF-α levels in serum are undetectable, but they increase in milk when the effects of intramammarily administered lipopolysaccharide on the acute-phase response in early and late lactation are examined (66).

Serum amyloid A, an apoprotein that is a component of high-density lipoprotein, is an acute phase protein (APP) (67). SAA functions by preventing platelet aggregation, phagocytosis, and the proliferation of lymphocytes and endothelial cells. Additionally, it promotes the production of prostaglandins and metalloproteinases as well as the migration of neutrophils and monocytes (68). Tumor necrosis factor-α, IL-1α, and IL-6 impact the transcription of SAA mRNAs (SAA1 and SAA2 isoforms), primarily in hepatocytes (69). Research on mastitis-affected cows has demonstrated that acute mammary infections cause a large (>200-fold) increase in SAA concentrations. In cows, SAA is thought to be the most sensitive APP, but it is not correlated with subclinical mastitis (70).

A variety of humoral and cellular immune response mechanisms interact through the transmission of stimulatory or inhibitory cytokine signals. An especially significant function is played by interleukin-6, which is thought to be the greatest stimulant of APP synthesis and secretion (46). Numerous cell types, including lymphocytes, monocytes, macrophages, endothelial cells, epithelial cells, and fibroblasts, express this cytokine, which is stimulated by bacteria. The concentrations of haptoglobin, ceruloplasmin, and transferrin are influenced by interleukin-6. Furthermore, it plays a role in T-cell antigen recognition activation, cytotoxic T-cell differentiation, and B-cell differentiation into distinct classes of Ig-releasing cells by acting as a stimulant (71).

## Conclusion

The findings of the present study suggested that supplementing lactating cows infected with mastitis improved immune indices without any adverse effects on rumen fermentation, blood chemistry, antioxidant capacity, and milk yield or milk composition. Additionally, further experiments are urged to examine the impacts of different doses of these supplements on ruminal microbial diversity and animal productive performance.

## Data Availability

The original contributions presented in the study are included in the article/supplementary material, further inquiries can be directed to the corresponding authors.

## Glossary

ALB: Albumin
ALP: Alkaline phosphatase
ALT: Alanine transaminase
AMS: Amylase
AST: Aspartate transaminase
Bas: Basophilic granulocyte
BUN: Blood urea nitrogen
CHE: Cholinesterase
CHOL: Total cholesterol
CRE: Creatinine
DBIL: Direct bilirubin
ECM: Energy corrected milk
Eos: Eosinophils
GGT: Gamma glutamyl transpeptidase
GLU: Glucose
GPx: Glutathione peroxidase
HCT: Hematocrit
HDL-c: High-density lipoprotein cholesterol
HGB: Hemoglobin
IgA: Immunoglobulin A
IgG: Immunoglobulin G
IgM: Immunoglobulin M
IL-1β: Interleukins 1β
IL-2: Interleukin 2
IL-6: Interleukin 6
IL-8: Interleukin 8
LAC: D-lactic acid
LDH: Lactate ehydrogenase
LDL-c: Low-density lipoprotein cholesterol
Lym: Lymphocytes
MC: *Macleaya cordata*
MCE: *Macleaya cordata* extract
MCH: Mean corpuscular-hemoglobin
MCHC: Mean corpuscular-hemoglobin concentration
MCV: Mean corpuscular volume
MDA: Malondialdehyde
Mon: Monocytes
MPV: Mean platelet volume
Neu: Neutrophils
NFS: Milk nonfat solid
PCT: Thrombocytocrit
PDW: Platelet distribution width
PLT: Platelet count
RBC: Red blood cell
RDW-CV: Red blood cell distribution width variable coefficient
RDW-SD: Red blood cell distribution width standard deviation
SCC: Somatic cell count
SOD: Superoxide dismutase
TAC: Total antioxidant capacity
TBIL: Total bilirubin
TG: Triglycerides
TNF-*α*: Tumor necrosis factor
TP: Plasma total protein
TS: Milk total solids
WBC: White blood cell
